# A unique blend of five human milk oligosaccharides supports recovery of infant microbiome composition and function after *ex vivo* antibiotic use

**DOI:** 10.3389/fped.2026.1765159

**Published:** 2026-03-05

**Authors:** Sinéad T. Morrin, Rachael H. Buck, David R. Hill

**Affiliations:** Abbott Nutrition, Columbus, OH, United States

**Keywords:** antibiotic resilience, human milk oligosaccharides, immune development, pediatric microbiome, pediatric nutrition

## Abstract

Human milk oligosaccharides (HMOs) are the third most abundant solid component of human breast milk, with well-established prebiotic and immunomodulatory functions. HMOs serve as selective substrates to support the growth of beneficial microbes in the developing gastrointestinal tract. At the same time individual HMOs have been shown to also exert selection against pathogens via direct anti-adhesive mechanisms. A longstanding hypothesis has held that HMOs act in concert and with other bioactive components of milk, and that this complex matrix of milk components collectively accounts for both the benefits to microbiome development and reduced risk of infectious disease associated with breastfeeding. The prebiotic activity of a diverse blend of fucosylated, acetylated, and sialylated HMOs was examined using microbiota cultured in an *ex vivo* model of the infant gastrointestinal tract before, during and after the supplementation of common childhood antibiotics. The anti-adhesive activity of this blend against infant-prevalent bacterial pathogens was tested using *in vitro* cultured intestinal epithelial cells. Taken together, this data suggests that a blend of 5 specific HMOs acts through multiple selection mechanisms to shape the development of the microbiota and interrupt opportunistic microbial pathogenesis.

## 1 Introduction

Human breast milk is nutritionally complete and optimally composed to support infant development and immune maturation. The World Health Organization, American Academy of Pediatrics, and other professional organizations overseeing pediatric nutrition recommend breastfeeding as the “gold-standard” nutrition for infants (1, 2). Breastfeeding is associated with both immediate and long-term health benefits, including reduced risks of infectious disease (3–5) and immune disorders, including allergy (6). Breast milk contains an abundance of bioactive macronutrients, micronutrients, hormones, growth factors and cytokines. This rich matrix of milk bioactives works in synchrony to support neonatal growth and immune maturation (7). The study of breastmilk and its bioactivity is of critical importance in the field of nutrition both as a source for infant formula fortification protocols as well as for advancing our general understanding of the factors shaping infant development.

The early-life developmental window represents a critical time for microbe–host interactions, with mounting evidence demonstrating that early establishment of the gastrointestinal microbiome is important for future health (8–10). Loss of key microbes or disturbance in microbial function alters the natural progression of the bacterial community and may have unwelcome consequences for host health (11, 12). Antibiotic use is common in childhood, with more than two-thirds of children receiving at least one course of antibiotics within the first 2 years of life (13). Despite the lifesaving necessity of antibiotic use, exposure to antibiotics in early life has been associated with increased risk of chronic disease (13–15). Specifically, infant exposure to antibiotics during treatment of infection is associated with microbial compositional changes, including an increase in the relative abundance of clostridia and a decrease in the relative abundance of bifidobacteria (16, 17).

Early exposure to antibiotics may also contribute to an increased risk of colonization by antimicrobial resistant bacteria (18, 19). Recent research suggests that the impact of antibiotics on the developing microbiome may contribute to chronic disease risk by interrupting the dynamic co-development of the microbiome and the immune system (20). Strategies to recover the disrupted microbiota are necessary to minimize antibiotic impact on both microbiota and immune development. Diet represents one of the leading factors influencing microbiome composition in infants (21) and numerous studies have compared the microbiome composition of breastfed and formula fed infants (22–24).

Over the past 20 years, human milk oligosaccharides (HMOs) have emerged as a major area of research in the field of pediatric nutrition (25–27). HMOs are defined by a core lactose disaccharide which is modified by additional mono- or polysaccharide structures, including fucose, sialic acid or acetylated carbohydrates. HMOs are generally categorized according to these terminal acetylated, fucosylated, or sialylated carbohydrate residues (28, 29). Altogether, HMOs are the third most abundant solid component of human breast milk yet do not constitute a direct source of calories. This apparent enigma was resolved by pioneering research that established robust prebiotic, antimicrobial, and immunomodulatory functions for these unique dietary carbohydrates (30–32).

Both preclinical and clinical studies demonstrate the impact of HMOs on the early infant gut microbiota (33, 34). HMOs serve as selective substrates to support the growth of beneficial microbes in the developing gastrointestinal tract (31, 35). This selectivity is achieved as a result of the terminal carbohydrate modifications, which resist digestion and absorption in the upper gastrointestinal tract (36–38). Consequently, HMOs arrive in the lower gastrointestinal tract intact and available for utilization by select constituents of the microbiota (31, 35, 39). Although a wide variety of microorganisms readily metabolize lactose, only *Bifidobacterium* and a select subset of *Lactobacillus* and *Bacteroides* species have evolved the enzymatic repertoire required to break HMOs into their constituent mono- and di-saccharides (40, 41). The gastrointestinal microbiota of many full-term healthy infants is relatively simplistic, dominated by the genus *Bifidobacterium* (42–44). *Bifidobacterium* is considered a foundational component of the microbiota which profoundly influences the intestinal environment and microbial community in early life (45–47)*. Bifidobacterium* abundance is correlated with healthy infant development as demonstrated through numerous pre-clinical and clinical studies (44, 48, 49). *Bifidobacterium* have the capacity to digest and consume the complete array of HMO structures utilizing a large repertoire of bacterial genes encoding an array of glycosidases and oligosaccharide transporters not found in other bacterial species (50–52). As a result, HMOs serve as a growth substrate reserved for key organisms associated with positive health outcomes in infancy.

In addition to supporting the growth and expansion of specific beneficial microbes, HMOs also play an active role in suppressing the establishment of infectious organisms within the gastrointestinal tract and respiratory tract (26). Early studies demonstrated that increased 2′-FL concentration in breastmilk was associated with reduced rates of *Campylobacter* induced diarrhea among breastfed infants (53). This led to experimental validation of the receptor decoy hypothesis using *in vitro* systems, which clearly demonstrated that purified 2′-FL isolated from human breastmilk competed for binding sites on the surface of *Campylobacter jejuni*, thereby preventing interaction between the pathogen and the intestinal epithelium (54). Numerous studies have since expanded upon these findings, demonstrating receptor decoy activity for numerous fucosylated, sialylated, and acetylated HMO structures against a wide range of bacterial, viral, fungal, and protozoan pathogens (25, 30, 32, 54–61).

A longstanding hypothesis has held that the rich blend of HMO structures found in breastmilk act in concert, and that the complex matrix of milk components collectively account for both the range and efficacy of immune protection against infectious disease and the expansion of beneficial microbes associated with breastfeeding (7, 25). These benefits include both the inhibition of gastrointestinal pathogens through receptor decoy activity, and the expansion of beneficial *Bifidobacteria* through selective prebiotic activity. The impact of a blend of 5 of the most abundant and widely prevalent (29, 62) HMOs on infant microbiome function and composition was evaluated before, during, and after *ex vivo* exposure to common childhood antibiotics as a measure of microbiome resilience to dysbiosis associated with infection. Additionally, the HMO blend was tested in experimental models *in vitro* to assess receptor decoy activity against common pediatric pathogens.

## 2 Materials and Methods

### 2.1 Materials

A mix of five human milk oligosaccharides (63) (Chr. Hansen HMO GmbH) were provided by Abbott Laboratories (Columbus, OH, USA). The human colonic adenocarcinoma cell line, HT-29 and human epithelial colorectal adenocarcinoma (Caco-2) were purchased from the American Type culture collection (ATCC). α-lactalbumin (α-LA) and cell culture reagents were purchased from Sigma-Aldrich (Wicklow, Ireland).

### 2.2 *In vitro* modeling of the gastrointestinal tract

#### 2.2.1 Study subjects

A total of 6 unique stool samples were collected from 6 healthy infants aged 4–6 months. All subjects had been exclusively formula fed for at least 30 days prior to participation in the study. Participating subjects had no prior history of antibiotic use or use of formula containing HMO. Samples were diluted in glycerol and aliquoted and stored at −80 °C within 2 h of defecation.

Fecal samples were collected by ProDigest, which is registered as a Biobank under EU and Belgian law with registration number BB220010. Fecal samples are continuously collected by ProDigest according to the ethical approval received from the Ethics Committee of Ghent University Hospital approval number ONZ-2022-0267.

#### 2.2.2 Bioreactor setup

A microbial bioreactor culture system was set up according to the methods described by Molly et al. (64). It consists of a succession of five reactors simulating the different parts of the human gastrointestinal tract. The first two reactors utilize the fill-and-draw principle to simulate different steps in food uptake and digestion, with peristaltic pumps adding a defined amount of feed (140 mL three times per day) and pancreatic and bile liquid (60 mL three times per day), respectively to the stomach and small intestine compartment and emptying the respective reactors after specified intervals. The last three compartments simulate the large intestine. These reactors are continuously stirred, and they have a constant volume and pH control. Retention time and pH of the different vessels are chosen to resemble *in vivo* conditions in the different parts of the colon. Upon inoculation with fecal microbiota, these reactors simulate the ascending, transverse and descending colon. Inoculum preparation, retention time, pH, temperature settings and reactor feed composition were previously described by Possemiers et al. (65). Upon stabilization of the microbial community in the different regions of the colon, a representative microbial community is established in the three colon compartments, which differs both in composition and functionality in the different colon regions.

#### 2.2.3 Experimental design

Each of the 6 unique infant stool samples were cultured in two parallel bioreactor systems corresponding to control and experimental treatment conditions. An initial stabilization period of 2 weeks followed the inoculation of the bioreactors systems. During this time the bioreactors were supplied with a basic nutritional matrix only, as described by Molly et al. (64). This allows for the maximum survival and stabilization of the fecal microbiota.

After the two-week stabilization period, control bioreactors were maintained using the basic nutritional matrix only and experimental bioreactors were treated with the basic nutritional matrix plus 2.5 g/L of a blend of HMOs with target concentrations of 2′-FL at 1.3 g/L, 3-FL at 0.325 g/L, LNT at 0.65 g/L, 3′-SL at 0.1 g/L, and 6′-SL at 0.125 g/L, reflecting proportions of 52%, 13%, 26%, 4%, and 5%, respectively. These treatments were maintained in parallel for 14 days.

Following the stabilization period and the initial two-week treatment, both control and experimental bioreactors were treated with amoxicillin:clavulanic acid at a concentration of 75 mg/day (25 mg/feeding cycle) for 7 days. This dose corresponded to the *in vivo* amoxicillin dose for children at 250 mg, three times per day (total 750 mg/day). Out of this total oral dose of amoxicillin, 90% is absorbed in the small intestine, resulting in only 10% of the dose entering the colon. Furthermore, clavulanic acid is generally co-administered in a clavulanic acid:amoxicillin ratio of 1:4 or 1:7 *in vivo*. Out of this oral dose of clavulanic acid, around 73% is absorbed in the small intestine, resulting in a clavulanic acid:amoxicillin ratio of 1:1.5 up to 1:2.6 in the colon. Therefore, a clavulanic acid:amoxicillin ratio of 1:2 was administered directly to the proximal colon during the experiment. Treatment of the control and experimental bioreactors with basic nutritional matrix or the nutritional matrix plus HMOs was continued during this period of antibiotic exposure.

After the cessation of antibiotic treatment, both control and experimental bioreactors were maintained for a further 14 days. During this time, treatment with basic nutritional matrix or the nutritional matrix plus HMOs was continued for the control, and experimental bioreactors, respectively.

#### 2.2.4 Bioreactor sampling and measurement of microbial metabolic activity

**Figure 1:**
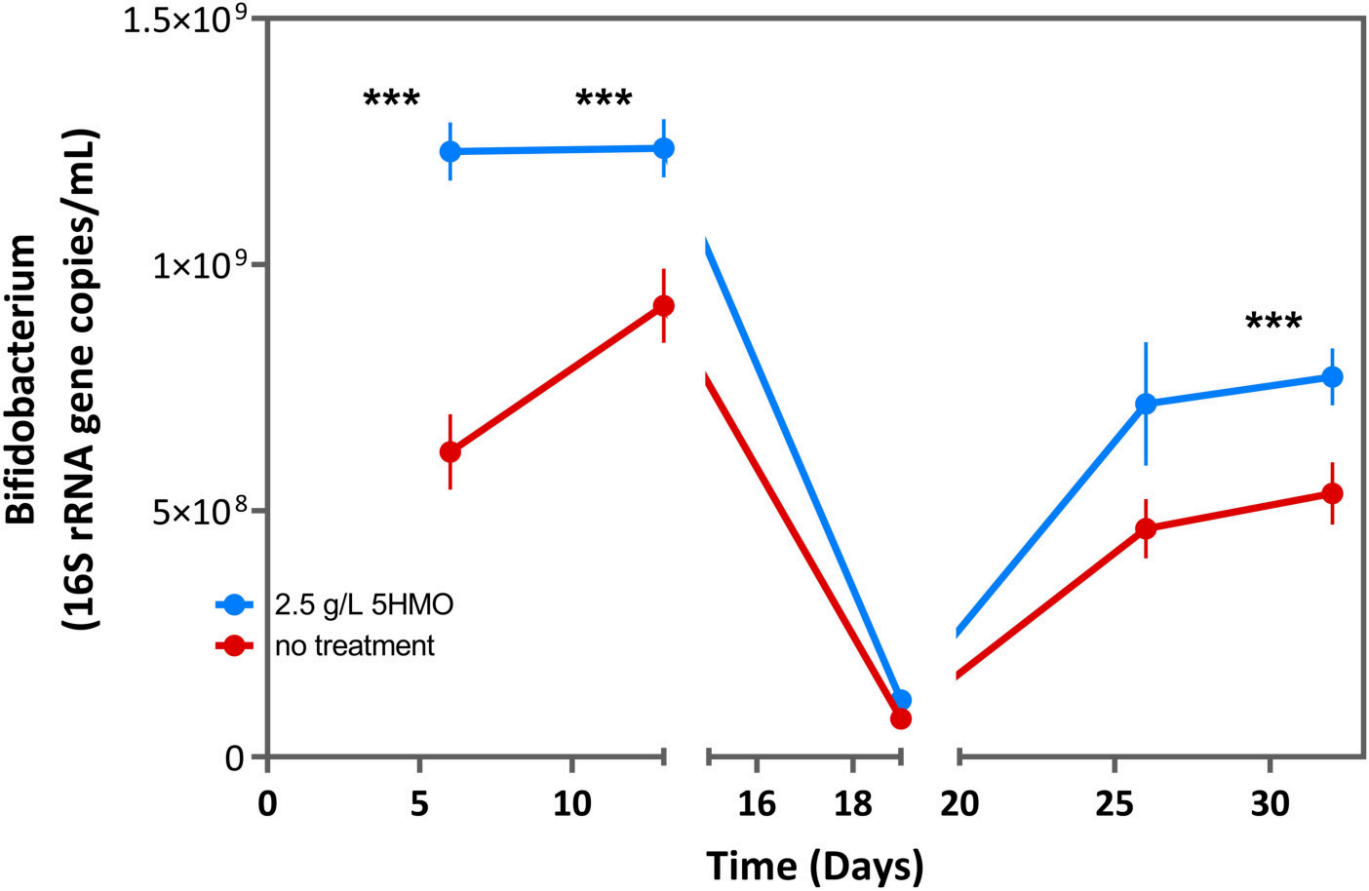
Total *Bifidobacterium* in infant microbiota treated with 2.5 g/L 5HMO blend or control media in SHIME bioreactor. Total *Bifidobacterium* (16S rRNA copies/mL) by day in cultures treated with 2.5 g/L 5HMO blend or control media. The antibiotic amoxicillin-clavulanate was administered on days 14–19. Data represents the mean of 6 independent infant microbiota cultures. *P* values are the result of two-tailed nonparametric Mann–Whitney test. *P* values ≤0.05 were considered statistically significant.

Both control and experimental bioreactors were sampled 3 times per week for overall microbial fermentative activity (acetate, propionate, butyrate, and total SCFA concentration). Additional samples were collected for quantification of *Bifidobacterium* via qPCR as indicated in Figure 1. SCFA production, including acetate, propionate, and butyrate was measured using the methods previously described by De Weirdt et al. (66). Ammonium measurement was performed as described by Van de Wiele et al. (67)

#### 2.2.5 Quantification of *Bifidobacterium*

*Bifidobacterium* quantification was performed using quantitative polymerase chain reaction (qPCR) on a StepOnePlus™ Real-Time PCR system (Applied Biosystems, Foster City, CA USA). Technical triplicates were evaluated for each sample. The qPCR for *Bifidobacterium* spp. as conducted as reported in (68).

### 2.3 Bacterial adhesion assays

#### 2.3.1 Bacterial strains and culture conditions

Bacterial strains used in this study and their respective growth media are listed in Table 1

**Table 1:** List of bacterial strains tested.

Strain	Media
*Listeria monocytogenes* NCTC 5348	Brain Heart Infusion
*Escherichia coli* ETEC O78:H11 ATCC 35401	Brain Heart Infusion
*Escherichia coli* EPEC O119 DSM 8699	Brain Heart Infusion

Bacterial strains were obtained from the American Type Culture Collection (ATCC), the Dairy Products Research Centre (DPC) culture collection (Teagasc Moorepark, Cork, Ireland) and Leibniz-Institut DSMZ (Braunschweig, Germany), respectively. Bacterial cultures were stocked in BHI broth containing 50% glycerol (v/v) and stored at −80 °C and propagated twice prior to use. All pathogenic strains were cultured 12–24 h before the adhesion assay (corresponding to 1 × 10^6^ CFU/mL) directly from a − 20 °C stock into brain heart infusion (BHI) (Oxoid® Ltd., Basingstoke, England) broth and left aerobically in a 37 °C incubator.

#### 2.3.2 Mammalian cell culture

The human colon adenocarcinoma cell line HT-29 and human epithelial colorectal adenocarcinoma (Caco-2) cell line were acquired from the American Type Culture Collection (ATCC). HT-29 cells were habitually cultured in McCoy's 5A modified medium (Merck) and Caco-2 cells were cultured in Dulbecco's modified Eagle's minimal essential medium DMEM (Merck) supplemented with 10% heat inactivated fetal bovine serum (Gibco, Invitrogen Corporation, USA), and 1% nonessential amino acids solution (Merck). Both cell lines were maintained in 75 cm^2^ tissue culture flasks and incubated at 37 °C in a humidified atmosphere (5% CO_2_). When the confluency of flasks reached 80%, the cells were passaged. The cells were fed every second day until 100% confluency was reached.

#### 2.3.3 Bacterial interaction assays

A series of bacterial interaction assays [adapted from Ross et al. (69)] were performed with HT-29 cells and all bacterial strains in the absence (control not-treated NT) and presence of 5HMOs, *α*-lactalbumin (α-LA) and combinations thereof. HT-29 cells were subsequently seeded into 12-well plates (Corning®, Lowell, Massachusetts, USA) at a concentration of 1 × 10^5^ cells/well and maintained at 37 °C in 5% CO_2_ in a humidified atmosphere. Prior to sample exposure, the cells were cultured and placed in McCoy's 5A modified medium supplemented with 2% (v/v) FBS 24 h beforehand. *Listeria monocytogenes* NCTC 5348, listed in Supplementary Table 1., was harvested at an optical density corresponding to 1 × 10^6^–× 10^8^ CFU/mL after 12–24 h (early stationary phase) from BHI broth, washed three times in phosphate buffered saline, pH 7.2 (PBS) and resuspended in McCoy's 5A modified medium supplemented with 0% FBS. Prior to exposure of the HT-29 cell monolayer, the bacterial strains were exposed to varying concentrations, individually and in combination with 5HMO and *α*-LA (0.5 g/L α-LA, 1 and 2.5 g/L 5HMO) for 1 h at 37 °C (5% CO2) in McCoy's 5A medium (0% FBS). The HT-29 confluent cell monolayers were then washed three times with PBS and exposed to 500 mL of the pre-incubated bacterial mix. HT-29 cells were then incubated for 1 h at 37 °C in a humidified atmosphere (5% CO2). After 1 h, the bacterial mix was removed from cells and the HT-29 cell monolayers were then washed three times with PBS and lysed with 500 mL of 0.1% Triton X-100 solution (Merck) in PBS for 45 min at 37 °C. and serially diluted as previously described and enumerated by spread-plating on BHI plates. Cell lysates were then serially diluted in maximum recovery dilutant (Oxoid®) and plated onto BHI agar (Oxoid®). The agar plates were incubated aerobically at 37 °C for 24 h and CFU were then counted.

#### 2.3.4 Host cell interaction assay

Host cell interaction assays [adapted from Ross et al. (69)] were performed with Caco-2 cells and bacterial strains *Escherichia coli* ETEC O78:H11 ATCC 35401 and *Escherichia coli* EPEC O119 DSM 8699 in the absence (control not-treated NT) and presence of 5HMO. Caco-2 cells were seeded into 24-well plates (Corning®, Lowell, Massachusetts, USA) at a concentration of 1 × 10^5^ cells/well and maintained at 37 °C in 5% CO2 in a humidified atmosphere. Prior to sample exposure, the cells were cultured and placed in Dublecco Modified Eagles Medium DMEM supplemented with 2% (v/v) FBS 24 h beforehand. The confluent monolayer of Caco-2 cells was washed twice in PBS and 500 mL of either 1 or 2.5 g/L 5HMO in McCoy's 5A media (2% FBS) were added to the wells for 1 h. The bacterial strains were harvested at an optical density corresponding to 1 × 10^7^ CFU/mL after 12–24 h (early stationary phase) from BHI broth, washed three times in phosphate buffered saline, pH 7.2 (PBS) and resuspended in DMEM supplemented with 0% FBS. The Caco-2 cells were then incubated together with the individual bacterial strains and 5HMO for 1 h at 37 °C. The Caco-2 cells were then incubated for 1 h at 37 °C in a humidified atmosphere (5% CO2). After incubation, Caco-2 cells were washed and lysed, and pathogens were plated out on agar as previously described above. All Caco-2 cell culture was performed by NIZO food research B.V. (Ede, Netherlands).

### 2.4 Statistical analysis

For the bioreactor studies, differences between the experimental treatment and the control treatment were tested using a non-parametric Mann-Whitney test in Prism version 9.4. A non-parametric test was utilized to account for non-normal distribution in baseline microbiota culture function and species composition. *P* values ≤0.05 were considered statistically significant. Adhesion assays were performed in triplicate and the percentage of inhibition was calculated by the equation: ((control-test)/control) × 100. Data are expressed as mean ± SD of the results of three independent assays conducted in triplicate. Graphs were generated using GraphPad Prism. ANOVA one-way were used, *p* ≤ 0.05 was considered significant. Synergy between *α*-LA and 5HMO was assessed using a Two-Way ANOVA with an interaction term on log10-transformed CFU; Type II and Type III tables are reported (Supplementary Tables 5a–b). In all figures “*” indicates *P* < 0.05; “**” indicates *P* < 0.01; “***” indicates *P* < 0.001.

## 3 Results

### 3.1 Addition of HMO blends to the infant microbiome increases *Bifidobacterium* density and enhances SCFA production in the SHIME bioreactor system

Utilizing the Simulator of the Human Intestinal Microbial Ecosystem (SHIME) bioreactor system, which stably and reproducibly recapitulates the dynamics of the fecal microbiome in a controlled *in vitro* experimental setting (70), the impact of supplementation with a blend of 5 HMOs was tested on 6 independent infant microbiome communities which were stably cultured in the SHIME bioreactor. This blend of HMOs contained representative acetylated (LNT), fucosylated (2′-FL, 3-FL), and sialylated (3′-SL, 6′-SL) structures, representing 5 of the most abundant HMOs found in breastmilk across global populations (29, 62). This blend has been clinically studied and associated with healthy growth and tolerance (71, 72) in infant formula as well as increases in stool *Bifidobacterium* (63, 73).

Following the two-week microbiome stabilization period, matched bioreactors containing inoculum from the same infant stool sample were treated with either 2.5 g/L of the 5HMO blend or control media alone once daily for 14 days. Luminal material was sampled from the colonic compartment of the bioreactor at multiple timepoints for the quantification of *Bifidobacterium* via qPCR (Figure 1; Table 2). Total *Bifidobacterium* density was increased nearly 2-fold in the 5HMO blend treated cultures relative to matched control cultures by day 6 (*P* = 2 × 10^−5^) and remained signifcantly elevated after 13 days in culture (*P* = 0.005). Thus, the addition of 2.5 g/L HMO blend was sufficient to increase baseline *Bifidobacterium* density in healthy infant microbiome bioreactor cultures.

**Table 2:** Total *Bifidobacterium* in infant microbiota treated with 2.5 g/L 5HMO blend or control media in SHIME bioreactor.

Day	Control treatment	2.5 g/L 5HMO	% change	*P* value
6	6.20E + 08	1.23E + 09	▴ 98.54%	**0**.**000002**
13	9.16E + 08	1.24E + 09	▴ 34.92%	**0**.**005035**
19	7.73E + 07	1.15E + 08	▴ 49.00%	0.123954
26	4.64E + 08	7.17E + 08	▴ 54.68%	0.261459
32	5.35E + 08	7.72E + 08	▴ 44.28%	**0**.**002752**

Total *Bifidobacterium* (16S rRNA copies/mL) by day in cultures treated with 2.5 g/L 5HMO blend or control media. The antibiotic amoxicillin-cavulinate was administered on days 14–19. Data represents the mean of 6 independent infant microbiota cultures. *P* values are the result of two-tailed nonparametric Mann–Whitney test. *P* values ≤0.05 were considered statistically significant.

**Figure 2:**
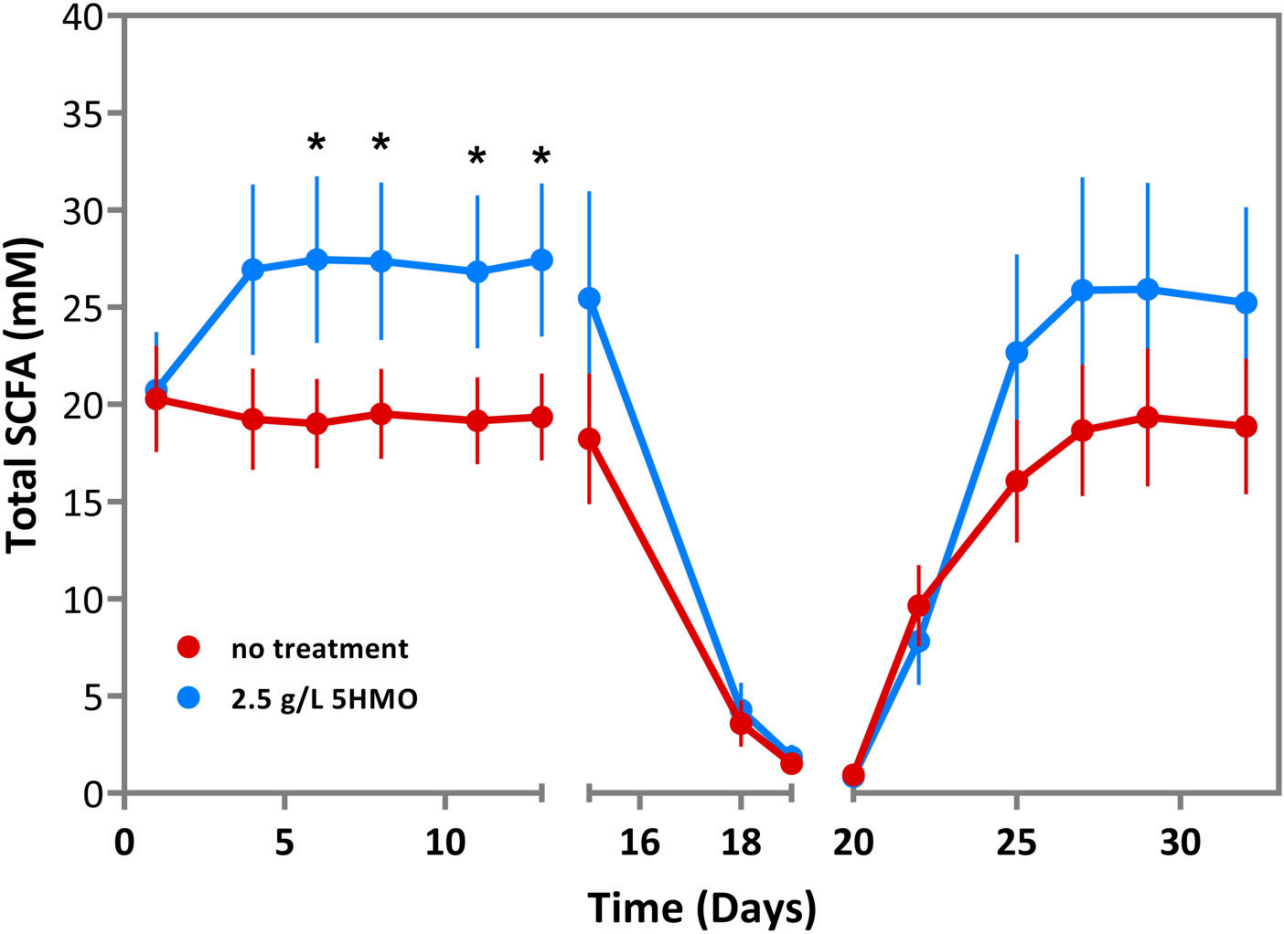
Total short-chain fatty acids (SCFA) in infant microbiota treated with 2.5 g/L 5HMO blend or control media in SHIME bioreactor. Total SCFA (mM) by day in cultures treated with 2.5 g/L 5HMO blend or control media. The antibiotic amoxicillin-clavulanate was administered on days 14–19. Data represents the mean of 6 independent infant microbiota cultures. *P* values are the result of two-tailed nonparametric Mann–Whitney test. *P* values ≤0.05 were considered statistically significant.

Luminal contents were further analyzed for the concentration of short-chain fatty acids (SCFAs) as a marker of beneficial microbial fermentation activity. During the first 14 days of bioreactor culture, total SCFA concentration was significantly elevated in 5HMO treated cultures relative to matched control cultures within 6 days of the initiation of HMO treatment (*P* = 0.029; Figure 2; Table 3). Total SCFA levels remained significantly elevated, with concentrations 40%–44% greater than matched control samples through 13 days of bioreactor culture (Figure 2; Table 3).

**Table 3:** Total short-chain fatty acids (SCFA) in infant microbiota treated with 2.5 g/L 5HMO blend or control media in SHIME bioreactor.

Day	Control treatment	2.5 g/L 5HMO	% change	*P* value
1	20.3	20.7	▴ 2.27%	0.962626
4	19.2	26.9	▴ 39.94%	**0**.**050859**
6	19.0	27.4	▴ 44.35%	**0**.**028958**
8	19.5	27.4	▴ 40.27%	**0**.**020464**
11	19.2	26.8	▴ 40.02%	**0**.**020464**
13	19.3	27.4	▴ 41.85%	**0**.**022359**
15	18.2	25.4	▴ 39.68%	0.180996
18	3.6	4.3	▴ 19.91%	0.541796
19	1.5	1.9	▴ 22.15%	0.562777
20	0.9	0.8	▾ −9.90%	0.839069
22	9.6	7.8	▾ −18.98%	0.191637
25	16.1	22.7	▴ 41.31%	0.388832
27	18.7	25.9	▴ 38.69%	0.461871
29	19.3	25.9	▴ 34.10%	0.323126
32	18.9	25.2	▴ 33.68%	0.170788

Total SCFA (mM) by day in cultures treated with 2.5 g/L 5HMO blend or control media. The antibiotic amoxicillin-cavulinate was administered on days 14–19. Data represents the mean of 6 independent infant microbiota cultures. *P* values are the result of two-tailed nonparametric Mann–Whitney test. *P* values ≤0.05 were considered statistically significant.

**Figure 3:**
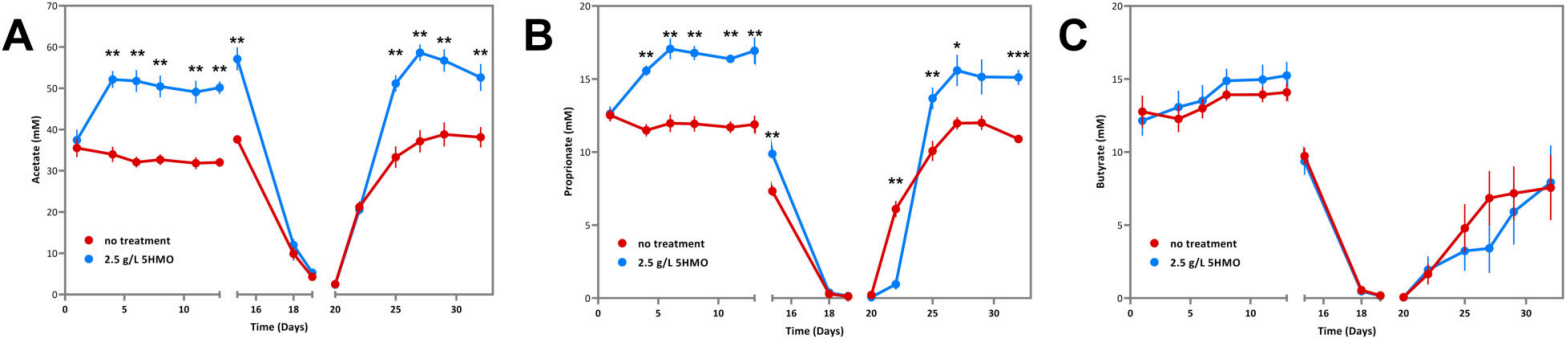
Individual SCFAs in infant microbiota treated with 2.5 g/L 5HMO blend or control media in SHIME bioreactor. (A) Acetate (mM) by day in cultures treated with 2.5 g/L 5HMO blend or control media. The antibiotic amoxicillin-clavulanate was administered on days 14–19. Data represents the mean of 6 independent infant microbiota cultures. *P* values are the result of two-tailed nonparametric Mann–Whitney test. **(B)** Proprionate (mM) by day in cultures treated with 2.5 g/L 5HMO blend or control media. The antibiotic amoxicillin-clavulanate was administered on days 14-19. Data represents the mean of 6 independent infant microbiota cultures. *P* values are the result of two-tailed nonparametric Mann–Whitney test. **(C)** Butyrate (mM) by day in cultures treated with 2.5 g/L 5HMO blend or control media. The antibiotic amoxicillin-cavulinate was administered on days 14–19. Data represents the mean of 6 independent infant microbiota cultures. *P* values are the result of two-tailed nonparametric Mann–Whitney test. *P* values ≤0.05 were considered statistically significant.

Total SCFA content is composed of multiple constituent structures: acetate, proprionate, and butyrate. An analysis of changes in the concentration of individual SCFAs revealed that increases in the total SCFA pool during the first 14 days of HMO supplementation were driven by increases in acetate (Figure 3A; Table 4) and proprionate (Figure 3B; Table 5), but not butyrate (Figure 3C; Table 6). Acetate concentration increased significantly by 52%–61% in HMO treated bioreactors relative to control bioreactors on days 4–15 (Figure 3A; Table 4). Similarly, proprionate concentration increased significantly by 35%–43% in HMO treated bioreactors relative to control bioreactors on days 4–15 (Figure 3B; Table 5). Despite these increases in acetate and proprionate, butyrate concentration did not vary significantly in the presence or absence of the HMO blend at any timepoint (Figure 3C; Table 6). These results suggest that 5HMO fortification supports overall microbial fermentation activity that results specifically in acetate and proprionate production.

**Table 4:** Acetate in infant microbiota treated with 2.5 g/L 5HMO blend or control media in SHIME bioreactor.

Day	Control treatment	2.5 g/L 5HMO	% change	*P* value
1	35.5	37.4	▴ 5.35%	0.588745
4	34.0	52.1	▴ 53.49%	**0**.**002165**
6	32.1	51.8	▴ 61.36%	**0**.**002165**
8	32.7	50.4	▴ 54.36%	**0**.**002165**
11	31.8	49.1	▴ 54.27%	**0**.**002165**
13	32.0	50.1	▴ 56.44%	**0**.**002165**
15	37.6	57.1	▴ 51.89%	**0**.**002165**
18	9.9	12.0	▴ 21.48%	0.484848
19	4.3	5.3	▴ 23.49%	0.393939
20	2.5	2.4	▾ −5.06%	0.818182
22	21.2	20.6	▾ −3.02%	0.699134
25	33.3	51.1	▴ 53.59%	**0**.**002165**
27	37.1	58.6	▴ 57.83%	**0**.**002165**
29	38.8	56.7	▴ 46.11%	**0**.**002165**
32	38.1	52.6	▴ 37.98%	**0**.**008658**

Acetate (mM) by day in cultures treated with 2.5 g/L 5HMO blend or control media. The antibiotic amoxicillin-cavulinate was administered on days 14–19. Data represents the mean of 6 independent infant microbiota cultures. *P* values are the result of two-tailed nonparametric Mann–Whitney test. *P* values ≤0.05 were considered statistically significant.

**Table 5:** Proprionate in infant microbiota treated with 2.5 g/L 5HMO blend or control media in SHIME bioreactor.

Day	Control treatment	2.5 g/L 5HMO	% change	*P* value
1	12.5	12.6	▴ 0.78%	0.699134
4	11.5	15.6	▴ 35.51%	**0**.**002165**
6	12.0	17.1	▴ 42.58%	**0**.**002165**
8	11.9	16.8	▴ 40.67%	**0**.**002165**
11	11.7	16.4	▴ 40.05%	**0**.**002165**
13	11.9	16.9	▴ 42.45%	**0**.**002165**
15	7.3	9.9	▴ 34.79%	**0**.**002165**
18	0.3	0.4	▴ 35.30%	0.093074
19	0.1	0.1	▴ 23.26%	0.24026
20	0.2	0.1	▾ −67.54%	0.937229
22	6.1	1.0	▾ −84.35%	**0**.**002165**
25	10.1	13.7	▴ 35.68%	**0**.**015152**
27	12.0	15.6	▴ 30.15%	**0**.**008658**
29	12.0	15.1	▴ 26.16%	**0**.**064935**
32	10.9	15.1	▴ 38.69%	**0**.**002165**

Proprionate (mM) by day in cultures treated with 2.5 g/L 5HMO blend or control media. The antibiotic amoxicillin-cavulinate was administered on days 14–19. Data represents the mean of 6 independent infant microbiota cultures. *P* values are the result of two-tailed nonparametric Mann–Whitney test. *P* values ≤0.05 were considered statistically significant.

**Table 6:** Butyrate in infant microbiota treated with 2.5 g/L 5HMO blend or control media in SHIME bioreactor.

Day	Control treatment	2.5 g/L 5HMO	% change	*P* value
1	12.8	12.1	▾ −4.84%	0.588745
4	12.3	13.1	▴ 6.59%	0.588745
6	13.0	13.5	▴ 4.00%	0.484848
8	13.9	14.9	▴ 6.86%	0.484848
11	13.9	15.0	▴ 7.44%	0.393939
13	14.1	15.2	▴ 8.14%	0.309524
15	9.7	9.4	▾ −3.84%	0.937229
18	0.6	0.5	▾ −15.86%	0.818182
19	0.2	0.2	▾ −11.02%	>0.999999
20	0.1	0.1	▾ −2.98%	0.699134
22	1.7	1.9	▴ 17.47%	0.818182
25	4.8	3.2	▾ −32.37%	0.484848
27	6.8	3.4	▾ −50.20%	0.093074
29	7.2	5.9	▾ −17.58%	0.484848
32	7.6	7.9	▴ 4.78%	>0.999999

Butyrate (mM) by day in cultures treated with 2.5 g/L 5HMO blend or control media. The antibiotic amoxicillin-cavulinate was administered on days 14–19. Data represents the mean of 6 independent infant microbiota cultures. *P* values are the result of two-tailed nonparametric Mann–Whitney test. *P* values ≤0.05 were considered statistically significant.

### 3.2 HMO blend mitigates reduction of *Bifidobacterium* populations during *ex vivo* antibiotic exposure and enhances recovery of microbiome function after the completion of antibiotic course

Additional experiments were conducted to assess the impact of a 5HMO blend on microbiome composition and function during and after antibiotic *ex vivo* exposure. Following two-weeks of culture with control media or media supplemented with 2.5 g/L of the HMO blend, the SHIME bioreactors were treated with a blend of the antibiotics, amoxicillin and clavulanic acid for 7 days as described above in the experimental methods. Amoxicillin-clavulanate is among the most frequently prescribed broad-spectrum antibiotics in children and is a first option for the treatment of otitis media, pneumonia, bacterial sinus infection, urinary tract infection and various skin and soft tissue infections (74). As anticipated, a notable decreases in *Bifidobacterium* density and SCFA production was observed following 7 days of antibiotic exposure (Figures 1–3). Both individual and total SCFA concentrations declined by >90%, indicating a broad suppression of microbial activity in the presence of antibiotics. The presence or absence of HMO supplementation had no significant impact on *Bifidobacterium* density or total SCFA production when antibiotics were present in the culture media. However, HMO supplementation significantly increased acetate and proprionate SCFAs during early antibiotic exposure (Figures 3A,B) compared to control cultures.

Antibiotic treatment was withdrawn from the SHIME bioreactor culture system after 7 days. Microbiome dynamics were monitored for a further 14 days to test for differences in the recovery of microbiome composition and function after antibiotic exposure. *Bifidobacterium* density recovered more rapidly in the presence of HMOs relative to control cultures, reaching densities comparable to untreated control conditions prior to antibiotic exposure within 2 weeks of antibiotic withdrawal (Figure 1; Table 2; *P* = 0.003). Similarly, total SCFA production recovered rapidly in the presence of 5HMOs, following the withdrawal of antibiotics. In HMO treated bioreactors, acetate production had recovered to pre-antibiotic levels within 5 days of the withdrawal of antibiotics and remained significantly elevated relative to control cultures for the duration of the bioreactor culture (Figure 3A; Table 4). Proprionate concentrations mirrored acetate in the post-antibiotic cultures, with concentrations reaching levels comparable to pre-antibiotic cultures within 5 days and remaining elevated for the duration of the experiment (Figure 3B; Table 5). In summary, the presence of 5HMOs was associated with an enhanced return to pre-antibiotic microbial composition and fermentation activity.

### 3.3 HMO supplementation reduces microbial ammonium generation in the SHIME bioreactor system

**Figure 4:**
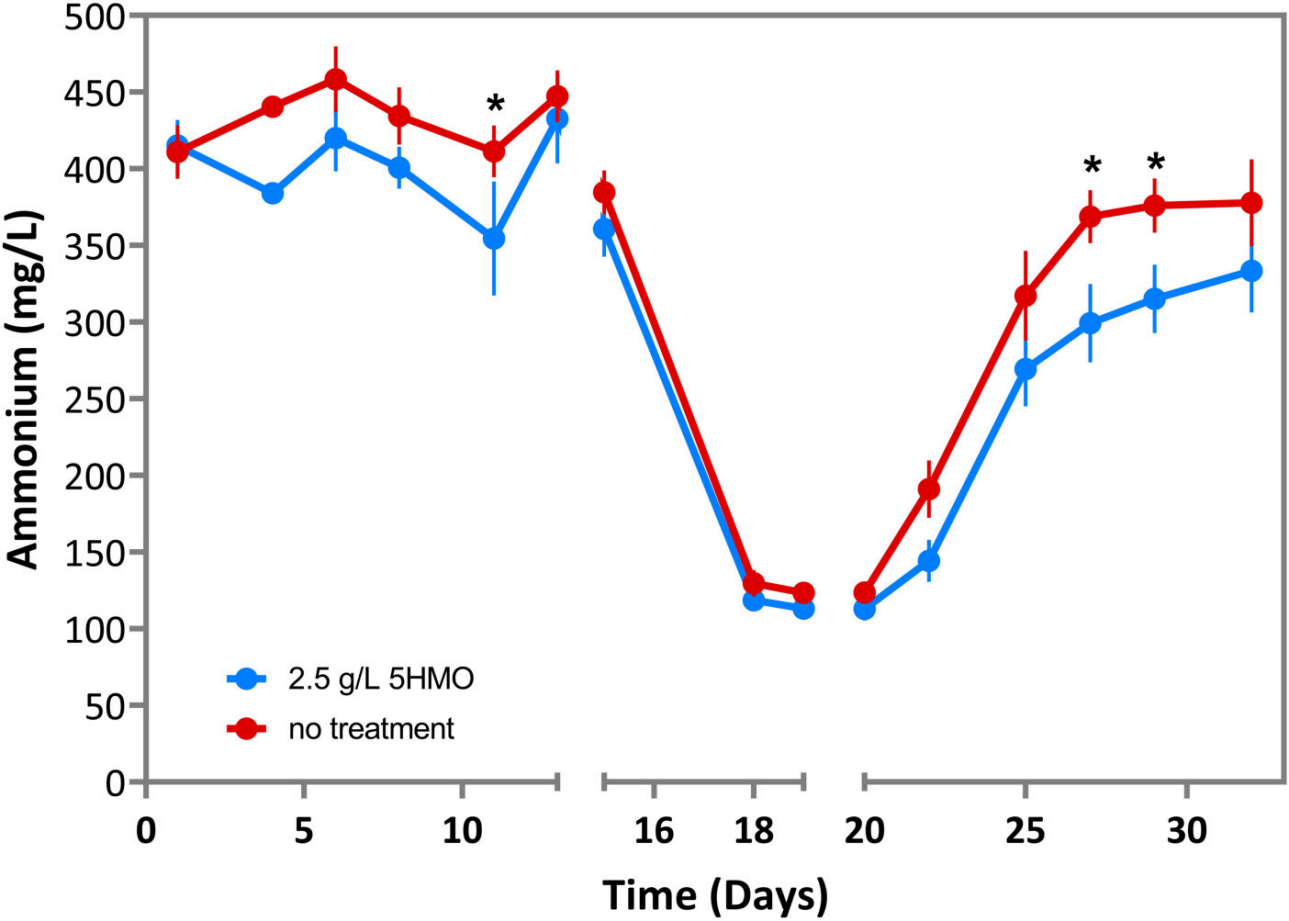
Ammonium in infant microbiota treated with 2.5 g/L 5HMO blend or control media in SHIME bioreactor. Ammonium concentrations by day in cultures treated with 2.5 g/L 5HMO blend or control media. The antibiotic amoxicillin-clavulanate was administered on days 14–19. Data represents the mean of 6 independent infant microbiota cultures. *P* values are the result of two-tailed nonparametric Mann–Whitney test. *P* values ≤0.05 were considered statistically significant.

The intestinal microbiota is a major source of ammonia, which is produced as a metabolic waste product during bacterial protein metabolism (75). The presence of 5HMOs generally suppressed ammonium generation in the SHIME bioreactor system, with significant reductions in ammonium observed on days 10 (*P* = 0.044, pre-antibiotic), 27 and 29 (post-antibiotics; *P* = 0.014 and *P* = 0.032, respectively; Figure 4).

### 3.4 Pre-treatment of human intestinal epithelial monolayers with 5HMO reduces adherence of pathogenic *E. coli*

**Figure 5:**
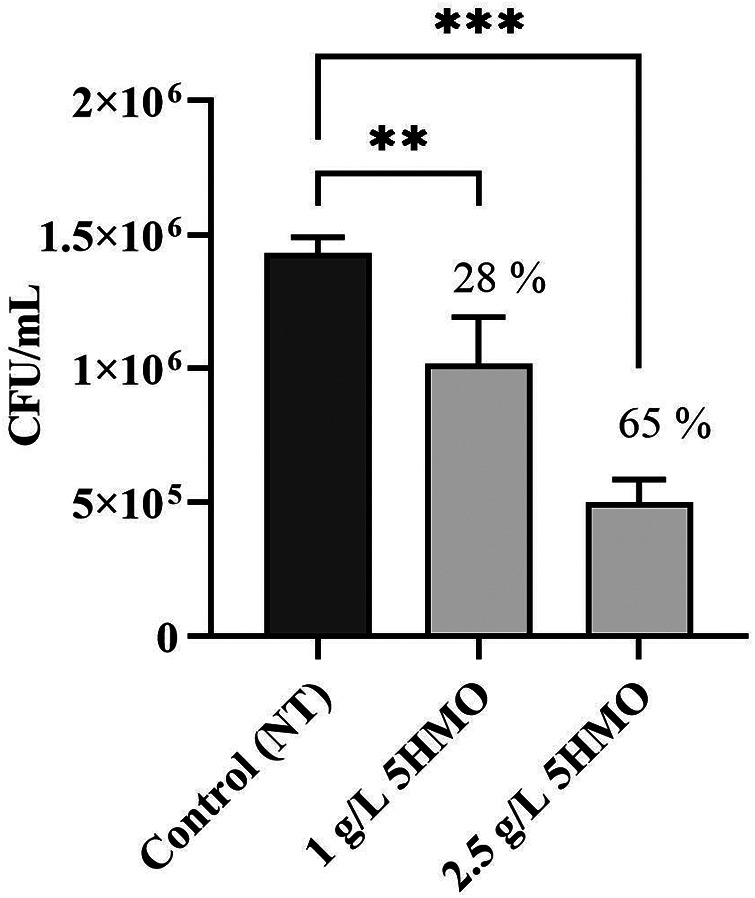
The effect of 5HMO fraction on association of *Escherichia coli* EPEC O119 DSM 8699 with caco-2 cells: effect of pre-incubating caco-2 cells with 5HMO prior to infection on the anti-infective activity (-, control; 1 g/L 5HMO, 2.5 g/L 5HMO); data are means ± standard deviation of 3 replicates; error bars represent standard deviation and an asterisk indicates values are significant (*P < 0.05, **P < 0.01, ***P < 0.01).

**Figure 6:**
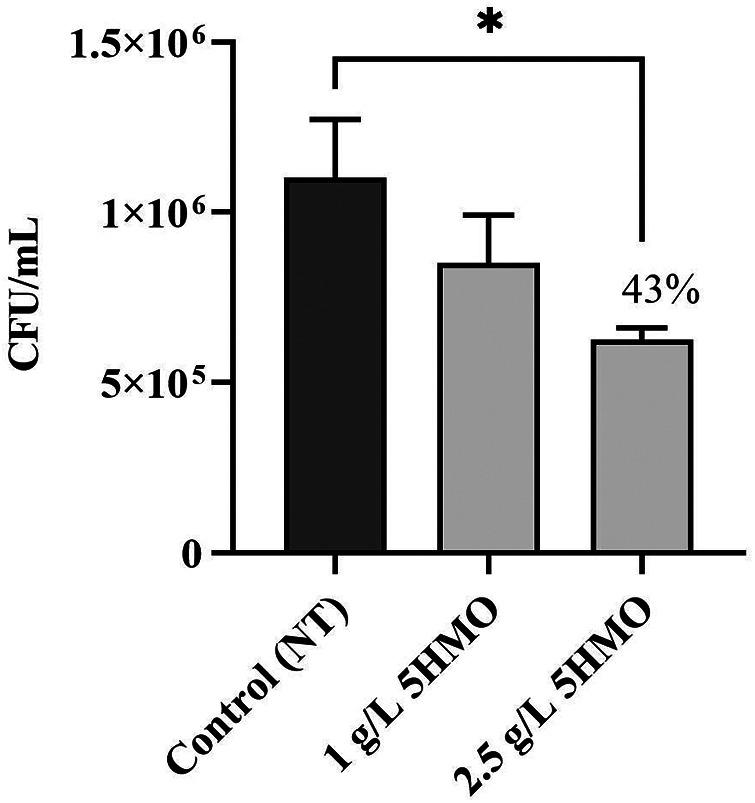
The effect of 5HMO fraction on association of *Escherichia coli* ETEC O78:H11 ATCC 35401 with caco-2 cells: effect of pre-incubating caco-2 cells with 5HMO prior to infection on the anti-infective activity (-, control; 1 g/L 5HMO, 2.5 g/L 5HMO); data are means ± standard deviation of 3 replicates; error bars represent standard deviation and an asterisk indicates values are significant (*P < 0.05, **P < 0.01, ***P < 0.01).

Previous studies have demonstrated that individual HMOs can stimulate changes in the expression of epithelial cell surface receptors that result in reduced pathogen adhesion in a cell culture system (76, 77). To test the hypothesis that a 5HMO blend acts similarly to stimulate anti-adhesive cell modulation, Caco-2 cells were pre-treated with the 5HMO blend or media alone for 1 h in culture prior to infection with pathogenic *E. coli* and subsequently measured the rate of bacterial adhesion. Pre-treatment with the HMO blend significantly reduced the adhesion of both enteropathogenic *E. coli* (EPEC, *P* < 0.001, Figure 5) and enterotoxigenic *E. coli* (ETEC, *P* < 0.05, Figure 6) in a dose-dependent manner at 1 h post-infection. Pre-exposure of human intestinal epithelial cells with the 5HMO blend was sufficient to substantially reduce adherence of two strains of a common gastrointestinal pathogen in culture.

### 3.5 Incubation of intestinal bacterial pathogens with the HMO blend reduces subsequent adhesion to intestinal epithelial monolayers

**Figure 7:**
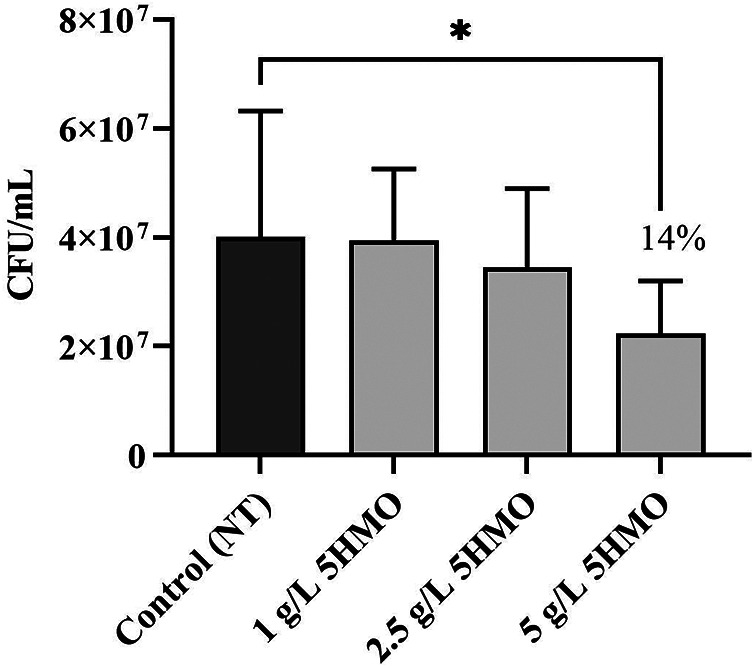
The effect of 5HMO fraction on association of *Listeria monocytogenes* NCTC 5348 with HT-29 cells: effect of pretreating *L.monocytogenes* with 5HMO on anti-infective activity. (control; 1 g/L 5HMO, 2.5 g/L 5HMO); Data are means ± standard deviation of 3 replicates; error bars represent standard deviation and an asterisk indicates values are significant (*P < 0.05, **P < 0.01, ***P < 0.01).

In addition to interactions with host cells, individual HMOs have been shown to interact directly with receptors on the surface of human pathogens. The hypothesis that prior exposure of bacteria to the 5HMO blend in solution inhibits subsequent bacterial adhesion to intestinal epithelial cells was tested. Our results demonstrate that 1 h pre-incubation of bacteria with the 5HMO blend in solution significantly inhibited the adherence of *Listeria monocytogenes* (*P* < 0.05, Figure 7) to intestinal epithelial monolayers in a dose-dependent manner. These results suggests that mixing HMO blends with pathogens in solution, such as may occur in the gastrointestinal tract during the early stages of infection, may result in interactions which partially neutralize the ability of the pathogen to adhere to host epithelia.

### 3.6 Presence of α-lactalbumin enhances the anti-adhesion activity of the HMO blend

**Figure 8:**
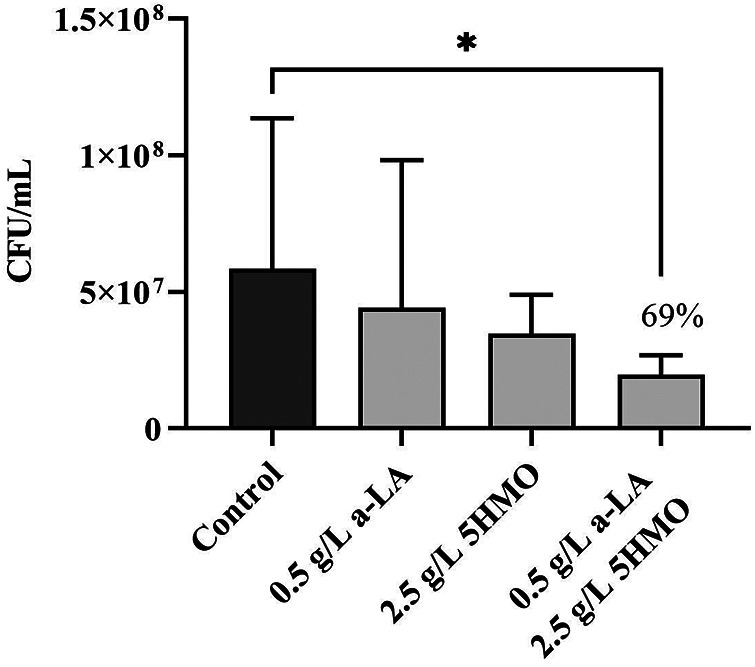
The effect of 5HMO and *α*-LA on association of *Listeria monocytogenes* NCTC 5348 with HT-29 cells: effect of pretreating *L. monocytogenes* with 5HMO and α-LA on anti-infective activity. (-, control; 0.5 g/L α-LA, 2.5 g/L 5HMO, a combination of 0.5 g/L α-LA and 2.5 g/L 5HMO); Data are means ± standard deviation of 3 replicates; error bars represent standard deviation, and an asterisk indicates values are significant (*P < 0.05, **P < 0.01, ***P < 0.01).

Within breast milk, bioactive milk proteins may work in synchrony with HMOs (7, 25, 26). Additional experiments were conducted which tested the hypothesis that an abundant milk protein, *α*-lactalbumin (*α*-LA), might modify the anti-infective properties of the 5HMO blend. *L. monocytogenes* (Figure 8) was incubated in solution with *α*-LA, the HMO blend, or a combination of *α*-LA with the HMO blend for 1 h prior to addition to epithelial monolayers in culture. The combination of the HMO blend with *α*-LA significantly reduced adhesion of *L. monocytogenes* relative to control treatment (*P* < 0.05, Figure 8). Notably, relatively low doses of the HMO blend (2.5 g/L) and α-LA (0.5 g/L) were required to achieve this effect compared to the higher dose of a HMO blend alone (5 g/L), which we had previously shown was required to significantly reduce *L. monocytogenes* adhesion (Figure 8**)**. Synergy between a-LA and 5HMO was assessed using a Two-Way ANOVA with an interaction term on log10-transformed CFU which was not significant, indicating no evidence of synergy; the combination is therefore described as showing enhanced activity relative to control due to an additive benefit. Thus, HMO and milk protein blends have additive direct anti-adhesive activity that is both dose and pathogen specific.

## 4 Discussion

This study characterized the effect of supplementation of a blend of five specific human milk oligosaccharides on the microbiota of healthy infants before, during, and after antibiotic exposure *ex vivo*. Our data demonstrate enhanced recovery of microbiome composition and function after antibiotic treatment in infant microbiota treated with an HMO blend relative to matched control microbiota (Figures 1–4). In addition, the impact of this HMO blend on both the bacterial and host cell response to common pathogenic organisms is described, demonstrating significant reduction in microbial adhesion in the presence of HMOs (Figures 5–8). These results provide evidence that specific HMO blends can shape the developing infant microbiota through both prebiotic and anti-adhesive mechanisms.

In this study, under healthy conditions (day 0–14), 5HMO significantly increased the abundance of *Bifidobacterium*. This data is consistent with the wealth of preclinical evidence demonstrating that HMOs serve as highly effective prebiotics to support *Bifidobacteria* expansion (31, 35, 52), and emerging clinical findings demonstrate that HMOs play a central role in supporting the robust growth of *Bifidobacteria* in the microbiome of infants (63, 73). In the first months after birth, the loss of *Bifidobacterium* species or disproportionate expansion of other competing bacteria, may significantly alter microbiome development and function (44, 45). Previous studies have observed antibiotic treatment in infants to be marked by a significantly increased abundance of Proteobacteria and decreased abundance of *Bifidobacterium* (78–80). Co-administration of 5HMOs during antibiotic therapy resulted in a marked recovery of *Bifidobacterium* as compared to the untreated control, with an additional stimulatory effect being observed when the application of 5HMOs was continued after antibiotic exposure (Figure 1). Our experimental findings are consistent with recent clinical evidence demonstrating that the addition of HMOs to infant formula results in increases in *Bifidobacteria* species (63, 73) and provide preclinical evidence that HMOs may promote retention and recovery of *Bifidobacteria* during antibiotic therapy.

Changes in the relative abundance of *Bifidobacterium* may subsequently alter the metabolic output of the microbiome. Short chain fatty acids, beneficial metabolites generated in the large intestine by *Bifidobacterium* and other anaerobic bacteria via fermentation of dietary carbohydrates, were also markedly improved in the 5HMO treated group (Figures 2, 3). SCFAs serve as an energy source for colonic epithelial cells, promote epithelial barrier integrity, stimulate mucus production, regulate fat deposition via G-protein-coupled receptors, and influence a range of immune functions (81, 82). Therefore, disruption to the normal production of SCFA due to microbiota dysbiosis caused by antibiotic treatment is associated with negative effects on host immunity and metabolism (83, 84). In this study, a significant increase in total SCFA was observed before antibiotic treatment (Figure 2). In the group supplemented with the mixture of 5 HMOs, SCFA production was restored after antibiotic cessation with significant increases observed in two important SCFA, propionate and acetate, compared to the control group (Figures 3A,B**)**. Healthy breastfed infants have higher levels of fecal acetate relative to formula fed infants (73), likely due to increased *Bifidobacteria*. Elevated intestinal propionate in the neonatal period has been shown to suppress the later onset of airway inflammation in preclinical studies and may help prevent bronchial asthma onset in human children (85, 86). The supplementation with the 5HMO mixture may help restore SCFA levels after antibiotic cessation to exert positive benefits on host health like that observed in the breast-fed infant.

To further understand the impact of 5HMO mitigating antibiotic-mediated disruption of the infant gut microbiota, ammonium production was measured (Figure 4). The intestinal tract is the primary source of ammonia and intestinal bacteria produce ammonia during protein catabolism (87). Although toxic at high concentrations, ammonia is converted to urea and excreted under normal physiological conditions (75). The 5HMO mixture significantly reduced ammonium levels before and after antibiotic treatment (Figure 4**)**, suggesting broad changes in microbiome protein catabolism when HMOs are present.

The host defense mechanisms of an infant can be classified as nonspecific (innate) or specific (acquired). Nonspecific mechanisms are effective without prior exposure to a microorganism or its antigens. Both individual structures and combinations of HMOs exhibit nonspecific immunity by inhibiting the pathogenesis of several bacterial and viral pathogens [reviewed (25)]. Several mechanisms of action have been proposed to account for the pathogenic deterrence capability of HMOs (28). HMOs may act as soluble decoy receptors for pathogens and pathogenic virulence agents due to their similarity to various cell surface glycan receptors. The pathogens bind to HMOs rather than to cell surface glycans, thereby blocking their adhesion to mucosal surfaces, a prerequisite to colonize the host and cause infection (28). Other established mechanisms by which pathogenic deterrence is accomplished include direct modulation of intestinal epithelial glycosylation (26, 76). The glycocalyx layer which decorates the intestinal surface is an anchoring point for pathogens and may be modulated in response to dietary components including HMOs. Modulation by HMOs has been previously shown to reduce the ability of enteropathogenic *E. coli* to adhere to the intestinal surface (76).

In this study, the unique combination of five HMO structures reduces pathogenic adherence by both direct and host-mediated mechanisms of pathogenic deterrence (Figures 5–8). The 5HMO combination significantly reduced both EPEC and ETEC adhesion to intestinal epithelial cells following prior incubation of epithelia with the HMO mixture. A 5HMO dose-dependent effect was observed with 2.5 g/L demonstrating a greater reduction in adherence than the lower HMO dose of 1 g/L. The exact mechanism by which the HMO combination modulates the cell surface to reduce subsequent pathogen adhesion remains to be deciphered. Previous *in vitro* studies have demonstrated that HMOs modulate expression of sialic acid and lactosamine epitopes (3′-SL) and intercellular adhesion molecule-1 (ICAM1) (2′-FL), producing a corresponding reduction in pathogen adhesion (76, 77). Reduced adherence of *Listeria monocytogenes* via HMO-intestinal cell modulation has been previously demonstrated (88); however, this is the first study that demonstrates a reduction in *L. monocytogenes* adhesion to an intestinal cell surface via decoy receptor activity. The unique combination of five HMOs studied here may have a broader anti-adhesive capacity than has been previously shown for individual HMO structures. Inhibitory effects via pre-incubation of *L. monocytogenes* with the 5HMO combination were further enhanced when combined with one of the dominant breast milk whey proteins, *α*-lactalbumin (Figure 8). Consistent with the statistical analyses, the combination did not demonstrate synergy but may indicate enhanced additive activity. α-Lactalbumin accounts for 28% of the total protein in human milk but only 3% of the total protein in bovine milk (89). It is therefore present at low concentrations in infant formula (90, 91). This study demonstrated the benefit of a low concentration of *α*-Lactalbumin in combination with five HMOs for reducing pathogen adhesion and, potentially, subsequent infection.

HMOs, as demonstrated in this study (Figures 5–7) and previous studies (30, 54, 77, 88), are anti-adhesive agents and reduce infection by inhibiting adherence to host cells and tissues, a prerequisite for many infectious diseases. HMOs may be highly beneficial dietary components for reducing the incidence of bacterial infections and subsequent antibiotic-mediated disruption of the gut microbiota (92). In contrast to traditional antibiotic agents which typically address a single targeted mechanism of action and have led to the proliferation and spread of resistant strains, specific HMO blends may play an important role in addressing antibiotic resistance due to their broad anti-adhesive and non-bactericidal mechanism of action and their application in multi-structure blends which act simultaneously to block multiple sites of adhesion (25, 26, 28). Further studies will enhance our understanding of the benefits of HMOs for curbing antibiotic resistance.

Overall, a blend of 5 specific HMOs has demonstrated significant benefits to microbiota fitness markers before, during and after antibiotic use in this *ex-vivo* model of the infant gut microbiota. Additional studies may further explore the impact of 5HMOs on the microbial ecology of the infant gut before, during and after antibiotic treatment. Questions of particular interest include characterization of the complete microbiota profile, particularly as our *in vitro* data demonstrates efficacy of the 5HMO mixture against common pathogenic families (Figures 5–8). The expansion of *Bifidobacterium* in the presence of HMOs may restructure multiple components of the microbiota. For example, the production of SCFAs by *Bifidobacteria* grown on HMOs contributes to lower intestinal pH, which in turn may inhibit the growth of bacterial pathogens such as *Clostridium difficile* (35, 83). Other evidence suggests that the exchange of metabolic products between HMO-fed *Bifidobacteria* and other beneficial microbes, a phenomenon known as cross-feeding, may support multiple beneficial organisms and reshape the community function in ways that benefit the human host (52, 93). The infant fecal samples used in this study had no prior antibiotic exposure. Therefore, the impact of repeated antibiotic exposure and the role of 5HMOs in the functional and compositional resilience of the infant microbiome remains to be addressed by future studies. Future studies may also examine the impact of complex blends containing additional milk proteins and other milk components for their ability to enhance the anti-adhesive and host modulatory properties of HMOs. Lastly, the effect of 5HMO and antibiotics on the microbiome markers of fecal samples from breast-fed donors and infants receiving complementary feeding is yet to be addressed.

The present study has several significant limitations that should be acknowledged. First, the relatively small sample size of 6 independent infant stool donors, although not inconsistent with previous preclinical reports (31), is not sufficient to reflect the broad global variability of infant microbiome diversity, genetic background, or feeding practices. Secondly, the cell culture models used do not fully recapitulate the complex physiology of the infant gastrointestinal tract *in vivo.* Future studies may seek to conduction additional verification in animal models of early life gastrointestinal development and in emerging *in vitro* models such as gastrointestinal organoids. Finally, while the *ex vivo* bioreactor model was applied to examine the impact of HMO supplementation over a 4 week period, with continued breastfeeding alongside complementary foods recommended until at least two years of age in alignment with World Health Organization (WHO) guidelines (2). Thus, the model system does not account for microbiome dynamics that may develop over longer periods of time or for the impact of early life HMO exposure after weaning. Future studies, including both animal models and clinical trials, may be able to examine these outstanding questions.

In conclusion, our study showed that supplementation of an *ex vivo* digestion model with 2.5 g/L blend of 5HMOs led to positive effects on the metabolic activity and composition of the fecal microbiota of 4–6 months old infants before, during, and after antibiotic exposure. Fermentation of 5HMOs resulted in an upsurge in SCFA with acetate and propionate significantly increased in the colonic bioreactor before, during and after antibiotic exposure in comparison to the non-supplemented group. All six infants in the 5HMO group were significantly colonized with *Bifidobacterium* compared to matched control samples and this remained true after antibiotic cessation and subsequent microbiome recovery. Finally, our results suggest that prebiotics, such as 5HMOs, might differentially alter microbial composition through various anti-adhesive mechanisms acting against common bacterial pathogens which may also lead to the increased SCFA and reduced ammonium. The 5 HMOs used in this study are representative of the major fucosylated, sialylated and acetylated HMO structures in breast milk. This *ex-vivo* and *in vitro* study suggests unique beneficial effects of 5 HMO supplementation in formula-fed infant populations.

## Data Availability

The raw data supporting the conclusions of this article will be made available by the authors, without undue reservation.
